# Association of plasma fatty acid-binding protein 3 with estimated glomerular filtration rate in patients with type 2 diabetes mellitus

**DOI:** 10.7150/ijms.66876

**Published:** 2022-01-01

**Authors:** Teng-Hung Yu, Chin-Feng Hsuan, Cheng-Ching Wu, Wei-Chin Hung, Thung-Lip Lee, I-Ting Tsai, Ching-Ting Wei, Jer-Yiing Houng, Fu-Mei Chung, Yau-Jiunn Lee, Yung-Chuan Lu

**Affiliations:** 1 Division of Cardiology, Department of Internal Medicine, E-Da Hospital, Kaohsiung 82445 Taiwan.; 2 Department of Emergency, E-Da Hospital, Kaohsiung 82445 Taiwan.; 3 Division of General Surgery, Department of Surgery, E-Da Hospital, Kaohsiung 82445 Taiwan.; 4 Division of Endocrinology and Metabolism, Department of Internal Medicine, E-Da Hospital, Kaohsiung 82445 Taiwan.; 5 School of Medicine, College of Medicine, I-Shou University, Kaohsiung, 82445 Taiwan.; 6 School of Medicine for International Students, College of Medicine, I-Shou University, Kaohsiung, 82445 Taiwan.; 7 Department of Biomedical Engineering, I-Shou University, Kaohsiung, 82445 Taiwan.; 8 Department of Electrical Engineering, I-Shou University, Kaohsiung, 82445 Taiwan.; 9 Department of Nutrition, College of Medicine, I-Shou University, Kaohsiung, 82445 Taiwan.; 10 Department of Chemical Engineering, I-Shou University, Kaohsiung, 82445 Taiwan.; 11 Division of Cardiology, Department of Internal Medicine, E-Da Dachang Hospital, Kaohsiung, 80794 Taiwan.; 12 Lee's Endocrinologic Clinic, Pingtung 90000 Taiwan.

**Keywords:** Type 2 diabetes mellitus, fatty acid-binding protein 3, estimated glomerular filtration rate

## Abstract

**Background:** Fatty acid-binding protein 3 (FABP3) located in renal mesangial and distal tubular cells, and had been shown to be a sensitive marker of renal injury, potentially be a mediator in pathogenesis of chronic kidney disease (CKD). Our previous study revealed that plasma FABP1 and FABP2 were independently associated with CKD, however, little is known about the relationship between plasma FABP3 level and CKD. The aim of this study was therefore to evaluate the plasma levels of FABP3 at different stages of estimated glomerular filtration rate (eGFR) in patients with type 2 diabetes mellitus (T2DM).

**Methods:** A total of 334 subjects with T2DM who enrolled in a disease management program were included in this study and stratified according to eGFR. Plasma FABP3 concentrations were measured by an enzyme-linked immunosorbent assay.

**Results:** FABP3 levels increased in parallel with the eGFR level. Increasing concentrations of FABP3 were independently and significantly associated with eGFR stage G2-G4. Age- and sex-adjusted FABP3 levels were positively associated with uric acid, urinary albumin-to-creatinine ratio, FABP1, FABP2, and fatty liver index, but negatively associated with eGFR and hemoglobin.

**Conclusion:** Our results indicate that circulating FABP3 in patients with T2DM is associated with eGFR, which suggests that increased plasma FABP3 may be involved in the pathogenesis of CKD.

## Introduction

Chronic kidney disease (CKD) is a chronic complication of diabetes, characterized by the presence of pathological quantities of urine albumin excretion and/or accompanied by a gradual deterioration in the glomerular filtration rate (GFR). It affects approximately 20-40% of patients with diabetes mellitus and is recognized as the most common cause of end-stage renal disease (ESRD) 1. Patients with CKD are at a high risk of cardiovascular disease and mortality and are associated with increased treatment costs 2. Although the pathogenesis of CKD remains unclear, evidence indicates that early recognition and interventions may delay the progression to ESRD and cardiovascular disease 3. Hence, early diagnostic markers for predicting and monitoring the progression of CKD are needed to enable the timely administration of the most appropriate protective treatments.

CKD in T2DM is characterized by unique glomerular injuries, including glomerular microaneurysm, Kimmelstiel-Wilson nodules, and mesangiolysis. Interestingly, nodular lesions have been shown to be comprised of lipid droplets 4, suggesting that lipid abnormalities may be involved in diabetic glomerular injury. Fatty acid-binding protein (FABP) functions as a long-chain fatty acid carrier in blood, and it plays an important role in lipid metabolism 5. Altered lipid metabolism, such as an increase in free fatty acids (FAs) and hyperlipidemia, has been shown to be an important characteristic of obesity and to contribute to kidney lesions 6. Intracellular FABPs are members of a multigene family encoding 15-kDa proteins which allow FAs to exit or enter the cellular cavity, and thereby play a role in cell injury and death induced by the FAs 7. Wu et al. showed that lipid dysmetabolism was involved in the development of obesity-related glomerulopathy, and that fatty acid-binding protein 3 (FABP3) (also known as heart-type fatty acid-binding protein or H-FABP) is especially up-regulated in the glomeruli 8. FABP3 is a member of the FABP family and is mainly expressed in the skeletal and heart muscles 9. Kimura et al. first demonstrated that FABP3 is present in human glomeruli, and that it is localized largely along the capillary walls 10. Subsequently, Chen et al. and Stieger et al. showed that FABP3 in the glomeruli is co-localized with podocytes, and that podocyte lesions play an important role in the development of many glomerular diseases 11,12. Furthermore, a previous animal study reported that FABP3 was induced in mesangial cells, and that it was likely to be a mediator of monocyte chemoattractant protein-1 (MCP-1) induction in diabetic kidney disease (DKD) 13. Moreover, Nauta et al. reported that urinary FABP3 was a marker of distal tubular damage, and that it was associated with eGFR independently of albuminuria, and therefore that it may be a promising urinary marker of DKD 14. In addition, our previous study revealed that plasma FABP1 and FABP2 were independently associated with CKD in T2DM 15, however, little is known about the relationship between plasma FABP3 level and CKD. The aim of this study was therefore to evaluate the plasma levels of FABP3 at different stages of estimated glomerular filtration rate (eGFR) in a Chinese population with T2DM.

## Methods

### Study participants

From January 2019 to December 2019, 334 consecutive patients with diabetes who visited the diabetic or cardiovascular clinics at E-Da Hospital were enrolled. The diagnosis of T2DM was based on the World Health Organization criteria 16. The mean age of the subjects was 67.1±9.8 years, and 69.4% were female. All of the study subjects were of Han Chinese origin, without any known ancestors of other ethnic origin, and they all lived in the same region at the time of the study. This study was approved by the Human Research Ethics Committee of Kaohsiung E-Da Hospital. Written informed consent was obtained from each participant before enrolment.

### Exclusion criteria

The exclusion criteria were: patients presenting with symptoms suggestive of type 1 diabetes, including acute presentation with heavy ketonuria (3+), diabetic ketoacidosis, or continuous requirement for insulin within 1 year of the diagnosis 17. Patients with liver cirrhosis, congestive heart failure, chronic lung diseases, urinary tract infections, urolithiasis, chronic otitis media, sinusitis, pelvic infections, chronic viral hepatitis, other known renal diseases, and eGFR <15 ml/min/1.73m^2^ were excluded on the basis of interviews, physical examinations, and urinalysis.

### Health examination protocol

Each patient received a detailed interview about his or her personal disease history and smoking history. Information on smoking habits was assessed using a standardized questionnaire. The patients' smoking status was classified as never having smoked, former smoker (ceased smoking for at least 1 year), or current smoker. In this study, former and current smokers were analyzed as a single group and compared to those who had never smoked. All patients underwent a complete physical examination and routine blood and urine biochemical analyses, and they were all assessed for the presence and extent of macrovascular or microvascular diabetic complications. Blood pressure was measured by a trained nurse using a digital automatic blood pressure monitor (model HEM-907; Omron, Omron, Japan) with the subject in a sitting position, after they had rested for 5 minutes. The fatty liver index (FLI) was calculated according to a previously published report by Bedogni et al. 18: FLI = [e^0.953^×log_e_ (triglycerides, TGs) + 0.139×body mass index (BMI) + 0.718×log_e_ (gamma-glutamyl transferase, GGT) + 0.053×waist circumference-15.745)] / [1+ e^0.953^×log_e_ (TGs) + 0.139×BMI + 0.718×log_e_ (GGT) + 0.053×waist circumference-15.745] ×100, with TGs measured in mmol/l, GGT in U/l, and waist circumference in cm.

### Anthropometric measurements

Body height was measured to the nearest 0.1 cm. Body weight was measured using an electronic scale to the nearest 0.1 kg with participants wearing a hospital gown. Waist and hip circumferences were measured to the nearest 0.1 cm at the narrowest point between the lowest rib and the uppermost lateral border of the right iliac crest. Hips were measured at their widest point. BMI was calculated by dividing the body weight in kilograms by the square of the body height in meters. The BMI and waist to hip ratio (WHR) were calculated for each subject.

### Laboratory measurements

Venous blood was drawn in the morning after an overnight fast, and plasma samples were kept at -80°C for subsequent assay. Serum creatinine was analyzed according to the kinetic Jaffé method on a SYNCHRON CX System analyzer (SYNCHRON, Los Angeles, CA) using reagents from Beckman (Beckman Coulter Diagnostic, Los Angeles, CA). Serum TGs, total cholesterol, low-density lipoprotein cholesterol (LDL-C), high-density lipoprotein cholesterol (HDL-C), albumin, hemoglobin, glucose, and white blood cell (WBC) count were determined using standard commercial methods on a parallel-multichannel analyzer (SYNCHRON, Los Angeles, CA). Hemoglobin A1c (HbA1c) was measured using high performance liquid chromatography. The concentrations of plasma FABP3 was determined using enzyme-linked immunosorbent assay (ELISA) kits (Invitrogen, Thermo Fisher Scientific Inc., USA). The dilution and standard curves were parallel, and the intra- and inter-assay coefficients of variation of the assays were 3.9% (n = 8) and 6.2% (n = 8), respectively, for FABP3. In addition, the concentrations of plasma FABP1 and FABP2 were determined using commercial ELISA kits (Cloud-Clone Corp., Katy, USA and R&D Systems, Inc., Minneapolis, USA). The analytical sensitivities were 0.59 ng/mL for FABP1 and 3.63 pg/mL for FABP2. ELISA was performed as per the instructions of the manufacturer. According to the manufacturer, the FABP1, FABP2, and FABP3 ELISAs had excellent specificity for the detection of human FABP1, FABP2, and FABP3, and no significant cross-reactivity or interference with analogues was observed. Samples were measured in duplicate in a single experiment.

### Renal function status definitions

Renal function (eGFR) was calculated using the four-variable Modification of Diet in Renal Disease (MDRD) study equation as eGFR = 175.0 × (serum creatinine^-1.154^) × (age^-0.203^) × 0.742 (if female). The eGFR categories were defined as stage 1, 2, 3a, 3b, 4, or 5 according to 2012 KDOQI definition 19.

### Statistical analysis

Data normality was analyzed using the Kolmogorov-Smirnov test. Continuous, normally distributed variables are presented as mean ± SD, and non-normally distributed variables as median (interquartile range). Statistical differences in variables were compared using one-way ANOVA for normally distributed variables followed by Tukey's pairwise comparisons. Categorical variables are presented as frequencies and/or percentages, and inter-group comparisons were analyzed using the chi-square test. Since the distributions of serum TGs, UACR, FLI, FABP1, FABP2, and FABP3 were skewed, logarithmically transformed values were used for the statistical analysis. Using multiple logistic regression, these variables were assessed for independent associations with the eGFR stage G2-G4. We further divided the distribution of FABP3 in pooled data into tertile and used general linear and logistic regression models to estimate the significant trends across increasing tertile and to estimate the odds ratios (ORs) of eGFR stage G2-G4 in each tertile using the lowest tertile as the reference category. Multivariate adjusted ORs are presented with 95% confidence interval (CI). Pearson's correlation coefficient and multiple linear regression analyses were used to examine correlations and independence between plasma FABP3 level and the values of other parameters. A p<0.05 was considered to be statistically significant. All data were analyzed using JMP version 7.0 for Windows (SAS Institute, Cary, NC, USA).

## Results

### Characteristics of the study subjects according to GFR categories

A total of 334 patients with T2DM were included in this cross-sectional study. The clinical and biochemical characteristics of the patients stratified by 2012 KDOQI eGFR categories are shown in Table 1. The prevalence rates of G1 (eGFR ≥90 ml/min/1.73m^2^), G2 (eGFR 60-89 ml/min/1.73m^2^), and G3a-G4 (59-15 ml/min/1.73m^2^) were 40.1%, 53.6%, and 6.3%, respectively. The patients with G3a-G4 had higher cardiovascular disease, oral hypoglycemic agent rates (OHA), age, diabetes duration, uric acid, estimated GFR, UACR, FABP1, and FABP3 than those with G1 and G2. Furthermore, the patients with G3a-G4 had higher creatinine, FABP2, and FLI than those with G1 (Table 1). Sex, hypertension, hyperlipidemia, retinopathy, neuropathy, treated with insulin alone, OHA + insulin use, statin use, angiotensin II receptor blocker (ARB) and angiotensin-converting enzyme inhibitor (ACEi) use, smokers, BMI, waist-to-hip ratio, systolic blood pressure (SBP), diastolic blood pressure (DBP), HbA1c, fasting glucose, total cholesterol, triglycerides, HDL-C, low-density lipoprotein cholesterol (LDL-C), white blood cell (WBC) count levels were the same among the three groups (Table 1).

### Associations between plasma FABP3 and eGFR stage G2-G4

Multivariate logistic regression analysis was performed to estimate the effects of plasma FABP3 level together with several other risk factors in the presence of eGFR stage G2-G4. The presence of eGFR stage G2-G4 was associated with plasma FABP3 level, age, OHA, and DBP (Table 2). Furthermore, increasing levels of FABP3 showed a significant linear trend and were independently associated with eGFR stage G2-G4, especially when concentrations were analyzed both by tertile and a continuous variable (Tables 2 and 3). Multiple logistic regression analysis in fully adjusted ORs in the second and third tertile were 1.98 (1.13-3.50) and 3.57 (1.97-6.60), respectively.

### Associations between plasma FABP3 levels and clinical laboratory data

Pearson's correlation analysis showed that plasma FABP3 levels were positively correlated with age, sex, SBP, uric acid, creatinine, UACR, FABP1, FABP2, FLI, and current smokers, but negatively correlated with eGFR and hemoglobin (Table 4). Furthermore, age- and sex-adjusted FABP3 levels were significantly positively associated with uric acid, UACR, FABP1, FABP2, and FLI, while they were significantly negatively associated with eGFR and hemoglobin. However, there were no significant associations among age and sex-adjusted FABP3 levels and BMI, WHR, SBP, DBP, fasting glucose, HbA1c, total cholesterol, TGs, HDL-C, LDL-C, creatinine, WBC count, platelet, and current smokers (Table 4).

## Discussion

In the present study, we demonstrated that plasma FABP3 levels were positively associated with uric acid, UACR, FABP1, FABP2, and FLI, and negatively associated with eGFR and hemoglobin, which increased in eGFR stage G2-G4 or even in a fully adjusted model. Our findings support the emerging hypothesis that FABP3 are associated with eGFR and comprises a component of FABP1 and FABP2 14,15. To our knowledge, this observation is the first that shows plasma FABP3 are associated with eGFR in patients with T2DM.

Previous studies have reported associations between increased urinary FABP3 concentrations and a deterioration in renal function in diabetic patients, and therefore that FABP3 concentration may be a promising urinary marker of DKD 14. The mechanisms behind the elevation of FABP3 in T2DM patients with CKD are not yet fully understood. An increase in serum FA accompanied with hyperglycemia is a known risk factor for the development of CKD in patients with T2DM. FA-induced MCP-1 induction has also been shown to be enhanced by an overexpression of FABP3 in mesangial cells. Consistently, an accumulation of macrophages and induction of MCP-1 expression have also been associated with FABP3 expression. It is possible that mesangial FABP3 can cause inflammation under diabetic conditions. Furthermore, it has been suggested that, during DKD, the accumulation of active macrophages is more evident in the kidney because of the elevation in chronic inflammation and oxidative stress, which consequently induce an increased expression of FABP3 13. Moreover, Nauta et al. reported that damage to distal renal tubules may result in both decreased glomerular filtration and increased tubular reabsorption, leading to an increase in FABP3 in the circulation 14. Thereby raising the possibility that FABP3 may link inflammation and oxidative stress that plays a role in the distal renal tubules damage and further contributes to CKD in patients with T2DM.

In the present study, we found that the levels of FABP3 were significantly higher in the patients with eGFR stage G3a-G4 than those with G1 and G2. Furthermore, our results showed that FABP3 level was significantly positively associated with uric acid and UACR, and negatively associated with hemoglobin and eGFR. This suggests that the concentration of plasma FABP3 increases with the progression of CKD. A number of epidemiological studies have reported an association between serum uric acid levels and the risk of DKD and DKD progression 20-22. Lipid accumulation is known to be related to renal damage 23 and hyperuricemia 24. A previous study also indicated that FABP3 was a possible marker linking lipid metabolism and renal damage 25. On the basis of these reports, we postulate that the effect of FABP3 on uric acid may be mediated by lipid metabolism and renal damage. In addition, as kidney disease progresses the prevalence of anemia increases, affecting nearly all patients with stage 5 CKD 26,27.

Our results showed that plasma FABP3 levels were positively associated with plasma FABP1 and FABP2. Of note, urinary FABP1 has been linked to CKD in patients with T2DM, and it has also been reported to be a predictor of progression to microalbuminuria in patients with type 1 diabetes 28,29. Furthermore, Okada et al. demonstrated that FABP2 can be used as a diagnostic and prognostic marker in patients with renal insufficiency 30. Moreover, our recent study suggested that FABP1 and FABP2 may be novel biomarkers of diabetic nephropathy 15. In the present study, we also observed increasing levels of FABP3 showed a significant linear trend and were independently associated with eGFR stage G2-G4. As previously reported, urinary FABP3 concentration was significantly elevated in normoalbuminuric diabetes patients compared to nondiabetic control subjects who had a similar eGFR. Consequently, the authors suggested that in addition to albuminuria, FABP3, as a marker of tubular damage, should also be used to predict the clinical outcomes of diabetic nephropathy 14. Furthermore, Pelsers reported that FABP3 appears to be a sensitive biomarker for the early detection of renal injury, and that its use may allow for better monitoring of patient treatment and kidney status 31. These facts suggest that FABP3 level could be a suitable biomarker for early detection with good value to detect CKD in patients with T2DM. Accordingly, clinicians and researchers should consider useful a multi-marker panel such as combination with FABP1, FABP2, and FABP3 to assess the severity of CKD in addition to measuring albuminuria.

Our study has also shown that plasma FABP3 concentrations are positively associated with FLI. Previously, Başar et al. showed that serum FABP3 concentrations increase in patients with NAFLD 32. Since patients with NAFLD exhibit multiple traditional and non-traditional risk factors (e.g. metabolic syndrome, increased C-reactive protein, interleukin-6, tumor necrosis factor-α levels and other acute-phase proteins, and so on) for CKD 33-35, it is not surprising that these patients also have a higher prevalence and incidence of CKD compared with those who do not have steatosis. The association of plasma FABP3 and FLI gives evidence that suggests plasma FABP3 has some role in inflammation and CKD. In addition, previous study found that plasma FABP3 are elevated in patients with peripheral artery disease, this fact indicated that plasma FABP3 be involved in the pathogenesis of diverse disease, other than diabetes and CKD 36.

In the present study, we have calculated eGFR using the four-variable MDRD study equation. In guidelines for the diagnosis and classification of CKD, KDOQI recommends estimation of GFR by the MDRD Study equation. Previous study 37 reported that limitations of the MDRD Study equation are that it has not been validated in children (age <18 years), pregnant women, the elderly (age >70 years), and racial or ethnic subgroups other than whites and blacks. The MDRD Study equation underestimates measured GFR in samples consisting primarily of persons with normal GFR 38. All GFR estimating equations are less valid in the non-steady state for creatinine (when serum creatinine is changing from day to day). Despite these limitations, GFR estimates using equations or nomograms are more accurate than serum creatinine alone. There are several limitations to this study. First, this was a single-center study, and the association of FABP3 with CKD needs to be confirmed in further multicenter studies. Second, this study has a cross-sectional design, and long-term, observational studies are needed to verify our results. Third, the number of enrolled patients was relatively small. However, this is a cross-sectional study, and cross-sectional studies are the best way to determine prevalence but do not allow for robust comparisons. Fourth, our analysis showed that only 33.3% and 9.5% of the included G3a and G4 patients had retinopathy and neuropathy respectively, and were lower than expected. This fact is resulted from that some of the subjects were referred to Endocrinology for targeted education consultations on nutrition and insulin administration technique from Departments of Nephrology and Cardiology. These patients often not underwent fully standardized complete complication survelence. In addition, it is important to study this prognostic value in diabetic patients with increasing FABP3 levels who do not yet have microalbuminuria, or GFR loss. Prospective cohort observation is undergoing to test the prognostic value of circulating FABP3 in the prediction of development or progression of CKD. Finally, whether increased FABP3 levels are associated with CKD requires further research.

## Conclusions

Our results indicate that circulating FABP3 in patients with T2DM is associated with eGFR, which suggests that increased plasma FABP3 may be involved in the pathogenesis of CKD.

## Figures and Tables

**Table 1:** Characteristics of the study subjects according to eGFR categories

Parameter	G1 (≥90)	G2 (60-89)	G3a-G4 (59-15)	p-value
N	134	179	21	
Age (years)	60.5±11.1	65.1±10.7	68.0±11.8	0.0002
Sex, female (n, %)	85(63.4)	92(51.4)	11(52.4)	0.098
Diabetes duration (years)	15.1±5.6	16.6±6.0	19.2±5.1	0.003
Hypertension (n, %)	12(9.0)	14(7.8)	0.(0.0)	0.363
Hyperlipidemia (n, %)	33(24.6)	63(35.2)	6(28.6)	0.130
Retinopathy (n, %)	29(21.6)	44(24.6)	7(33.3)	0.583
Neuropathy (n, %)	11(8.2)	16(8.9)	2(9.5)	0.953
Cardiovascular disease (n, %)	19(14.2)	36(20.1)	11(52.4)	0.0002
OHA (n, %)	125(93.3)	164(91.6)	14(66.7)	0.0004
Insulin alone (n, %)	26(19.4)	46(25.7)	3(14.3)	0.272
OHA + insulin (n, %)	23(17.2)	37(20.7)	1(4.8)	0.186
Statin use (n, %)	107(79.9)	151(84.4)	17(81.0)	0.577
ARB and ACEi use (n, %)	57(42.5)	87(48.6)	12(57.1)	0.348
Smokers (n, %)	28(20.9)	51(28.5)	8(38.1)	0.136
Body mass index (kg/m^2^)	25.3±4.6	25.1±3.8	25.2±3.1	0.946
Waist-to-hip ratio	0.84±0.23	0.87±0.21	0.82±0.28	0.145
Systolic blood pressure (mmHg)	134±17	134±17	140±23	0.404
Diastolic blood pressure (mmHg)	77±11	78±10	80±13	0.427
HbA1c (%)	8.7±2.0	8.4±1.9	9.3±2.6	0.120
Fasting glucose (mg/dl)	155.9±52.8	154.0±48.0	158.2±39.6	0.692
Total cholesterol (mg/dl)	190.9±39.9	187.6±37.2	182.7±31.4	0.569
Triglycerides (mg/dl)	128.8(81.0-148.5)	136.7(76.0-167.0)	136.0(74.5-180.5)	0.737
HDL cholesterol (mg/dl)	50.5±13.0	47.6±13.5	49.0±12.5	0.168
LDL cholesterol (mg/dl)	109.8±34.3	108.2±30.5	101.3±31.9	0.530
Uric acid (mg/dl)	4.9±1.4	5.3±1.6	6.1±2.3	0.004
Creatinine (mg/dl)	0.8±0.2	0.9±0.2	0.9±0.2	0.001
Estimated GFR (ml/min/1.73m^2^)	102.3±12.8	77.4±7.8	44.8±13.5	<0.0001
UACR (mg/g)	70.8(8.3-39.3)	66.2(6.8-45.7)	261.7(8.7-174.0)	0.001
White blood cell (10^9^/l)	6.379±1.677	6.745±1.732	7.019±1.908	0.204
FABP1 (ng/ml)	16.1(7.3-20.0)	19.5(8.4-25.2)	42.2(14.4-36.3)	<0.0001
FABP2 (ng/ml)	1.7(1.2-2.2)	2.4(1.4-2.6)	3.0(1.8-2.9)	0.040
FABP3 (ng/ml)	1.3(0.8-1.4)	1.6(1.0-1.8)	4.3(1.5-6.4)	<0.0001
Fatty liver index	33.6(11.0-48.5)	34.3(12.0-52.5)	38.2(17.1-68.0)	0.042

Data are presented as mean ± SD, frequency (percent), or median (interquartile range). OHA, oral hypoglycemic agent; ARB, angiotensin II receptor blocker; ACEi, angiotensin-converting enzyme inhibitor; HDL, high-density lipoprotein; LDL, low-density lipoprotein; GFR, glomerular filtration rate, UACR, urinary albumin-to-creatinine ratio; FABP, fatty acid-binding protein.

**Table 2:** Multiple logistic regression analysis with estimated glomerular filtration rate stage G2-G4 as the dependent variable

	exp(B)	95% Confidence Interval	p-value
Age	1.05	1.02-1.08	0.001
Sex	1.25	0.68-2.31	0.471
Cardiovascular disease	1.32	0.65-2.70	0.441
Oral hypoglycemic agent	0.25	0.09-0.74	0.012
Smoking	1.10	0.55-2.20	0.795
BMI	0.99	0.93-1.07	0.928
Systolic BP	0.98	0.97-1.00	0.054
Diastolic BP	1.04	1.00-1.09	0.031
Fasting glucose	0.99	0.99-1.00	0.535
Total cholesterol	0.99	0.97-1.01	0.383
Triglyceride	1.00	0.99-1.01	0.610
HDL-cholesterol	0.99	0.96-1.02	0.614
LDL-cholesterol	1.01	0.99-1.03	0.401
Log FABP3	7.76	2.12-28.47	0.002

BMI, body mass index; BP, blood pressure; HDL, high-density lipoprotein; LDL, low-density lipoprotein, FABP, fatty acid-binding protein.

**Table 3:** Univariate and multivariate analysis of the impact of plasma fatty acid-binding protein 3 level on eGFR stage G2-G4

Factor	Tertiles of FABP3			
	Q1 (95% CI)	Q2 (95%CI)	Q3 (95%CI)	*P* value
**All subjects**				
No. of cases/reference	51/62	68/43	81/29	<0.0001
Cut off FABP3 concentration (ng/mL)	<1.02	1.02-1.53	1.53-15.19	
Univariate	1.00	1.92 (1.13-3.29)	3.40 (1.95-6.03)	<0.0001
Multivariate^a^	1.00	1.98 (1.13-3.50)	3.57 (1.97-6.60)	<0.0001

Values shown are cut-offs of plasma FABP3 levels of all subjects, and odds ratios (ORs) with 95% confidence intervals (CIs). ^a^ Adjusted for body mass index, HbA1c, systolic blood pressure, diastolic blood pressure, high-density lipoprotein, low-density lipoprotein, triglycerides, cardiovascular disease, oral hypoglycemic agent, and smoking status. FABP, fatty acid-binding protein.

**Table 4:** Associations between plasma fatty acid-binding protein 3 levels and clinical laboratory data

	Model 1		Model 2	
	r	p-value	β	p-value
Age	0.337	<0.0001	-	-
Sex	0.159	0.004	-	-
Body mass index	-0.028	0.610	-0.020	0.710
Waist-to-hip ratio	0.099	0.072	0.055	0.281
SBP	0.122	0.025	0.012	0.827
DBP	0.040	0.471	-0.004	0.939
Fasting glucose	0.034	0.541	0.040	0.434
HbA1c	0.070	0.203	0.062	0.222
Total-cholesterol	-0.028	0.610	-0.030	0.559
Triglycerides	0.093	0.089	0.076	0.135
HDL-cholesterol	-0.057	0.302	-0.006	0.915
LDL-cholesterol	-0.081	0.138	-0.086	0.089
Uric acid	0.382	<0.0001	0.339	<0.0001
Creatinine	0.123	0.025	0.010	0.877
Estimated GFR	-0.366	<0.0001	-0.275	<0.0001
UACR	0.213	<0.0001	0.190	<0.0001
White blood cell count	-0.042	0.448	-0.011	0.828
Platelet	-0.082	0.135	0.063	0.248
Hemoglobin	-0.117	0.032	-0.252	<0.0001
FABP1	0.483	<0.0001	0.437	<0.0001
FABP2	0.357	0.0002	0.361	<0.0001
Fatty liver index	0.122	0.028	0.106	0.042
Current smoking	0.130	0.017	0.081	0.206

Model 1: Pearson correlation coefficient. Model 2: Regression coefficient adjusted for age and sex. Abbreviations: SBP, systolic blood pressure; DBP, diastolic blood pressure; HDL, high-density lipoprotein; LDL, low-density lipoprotein; GFR, glomerular filtration rate; UACR, urinary albumin-to-creatinine ratio; FABP, fatty acid-binding protein.
